# Analyzing the Importance of Driver Behavior Criteria Related to Road Safety for Different Driving Cultures

**DOI:** 10.3390/ijerph17061893

**Published:** 2020-03-14

**Authors:** Danish Farooq, Sarbast Moslem, Rana Faisal Tufail, Omid Ghorbanzadeh, Szabolcs Duleba, Ahsen Maqsoom, Thomas Blaschke

**Affiliations:** 1 Department of Transport Technology and Economics, Budapest University of Technology and Economics, 1111 Budapest, Hungary; farooq.danish@mail.bme.hu (D.F.); moslem.sarbast@mail.bme.hu (S.M.); duleba.szabolcs@mail.bme.hu (S.D.); 2 Department of Civil Engineering, Comsats University Islamabad, Wah Cantt 47040, Pakistan; faisal.tufail@ciitwah.edu.pk (R.F.T.); ahsen.maqsoom@ciitwah.edu.pk (A.M.); 3 Department of Geoinformatics, University of Salzburg, 5020 Salzburg, Austria; thomas.blaschke@sbg.ac.at

**Keywords:** driver behavior criteria, fuzzy analytic hierarchy process, pairwise comparison, ranking, traffic cultures, concordance, road safety

## Abstract

Driver behavior has been considered as the most critical and uncertain criteria in the study of traffic safety issues. Driver behavior identification and categorization by using the Fuzzy Analytic Hierarchy Process (FAHP) can overcome the uncertainty of driver behavior by capturing the ambiguity of driver thinking style. The main goal of this paper is to examine the significant driver behavior criteria that influence traffic safety for different traffic cultures such as Hungary, Turkey, Pakistan and China. The study utilized the FAHP framework to compare and quantify the driver behavior criteria designed on a three-level hierarchical structure. The FAHP procedure computed the weight factors and ranked the significant driver behavior criteria based on pairwise comparisons (PCs) of driver’s responses on the Driver Behavior Questionnaire (DBQ). The study results observed “violations” as the most significant driver behavior criteria for level 1 by all nominated regions except Hungary. While for level 2, “aggressive violations” is observed as the most significant driver behavior criteria by all regions except Turkey. Moreover, for level 3, Hungary and Turkey drivers evaluated the “drive with alcohol use” as the most significant driver behavior criteria. While Pakistan and China drivers evaluated the “fail to yield pedestrian” as the most significant driver behavior criteria. Finally, Kendall’s agreement test was performed to measure the agreement degree between observed groups for each level in a hierarchical structure. The methodology applied can be easily transferable to other study areas and our results in this study can be helpful for the drivers of each region to focus on highlighted significant driver behavior criteria to reduce fatal and seriously injured traffic accidents.

## 1. Introduction

The Global status reported a high number of road traffic deaths per year, approximately 1.35 million [1]. The situation analysis reports that major casualties are due to human-related issues, therefore handling them becomes the highest dynamic target of road safety actions [2]. Human factors have been estimated to be a sole or leading causal factor in approximately 90% of road traffic accidents [3,4,5]. The previous study focused on road safety primary factors. Driving behavior, driver risk perception and experience were the fundamental factors that impacted road safety [6].

There are usually significant differences in driving practice between countries [7]. The driver behavior significantly varies from country to country with different traffic risk perception [8]. A comparison study was conducted to investigate the perceived risks variations of traffic accidents in various countries e.g., North American and Japan. The results indicated that participants from Japan projected a higher risk of traffic accidents than participants from North American [9]. The cultural differences were evaluated in risk perception, driver behavior and traffic safety approaches in Norway and Ghana. Higher traffic risk was observed for younger age groups as compared to older ones in both countries [10]. 

Naturalistic driving is a recently employed research method, studying road users’ everyday driving situation, instead of in a scientific experiment. For a long time, controlled experiments such as self-reporting questionnaires, driving simulators were the basic method of examining driving behavior. The main advantage of this type of experiment is the huge degree of control over the variables that (may) influence driving behavior. However, controlled experiments are very often performed in a designed environment. This causes the transfer of the results to real traffic more difficult [11,12]. Within traffic studies with specific goals to study driver behavior, the scientific investigation related to naturalistic driving (ND) examination has increasingly gained significance in the last years [13,14].

Several studies have used multi-criteria decision-making applications to evaluate human behavior [15,16,17]. A review of road safety models in the literature has shown that several studies have proposed approaches based on multi-criteria decision-making analysis to calculate road safety problems [18,19,20,21]. Analytic Hierarchy Process (AHP) was the best approach to prioritize suburban road safety indicators to access the factors that can decrease traffic accidents as well as the severity of accidents in Iran [22]. Regardless of the benefits of the multi-criteria decision-making method of the AHP, this method is usually subject to some inabilities [23]. The AHP method prioritization may not be accurate because of the subjective judgment by perception; evaluation, improvement and selection that is solely the preference of decision-makers have a great influence on the output of AHP. Moreover, some of the participants who are asked to fill the questionnaires may not be completely conscious of the significance of some of the indicators [24]. The inconsistency and the associated uncertainty may be increased for that current research when participants compare the driver behavior indicators beyond indicators related to road safety. 

To manage these tricky problems, some techniques have been used along with the AHP to minimize the associated uncertainty and the inconsistency, such as using the inter connections [25], frequency ratio [26,27,28], sensitivity and uncertainty analysis [29], interval calculations [24], modified analytical hierarchy process [30], weights-of-evidence bivariate statistical model [31]. However, many researchers integrate fuzzy theory with AHP to deal with the associated uncertainty within the comparisons [32,33,34,35,36,37]. The Fuzzy Analytic Hierarchy Process (FAHP) is a more precise technique as compared to AHP. AHP technique lacks in the human thinking behavior area which is more precise in the case of FAHP. So, FAHP can be considered as more accurate as compared to AHP in terms of human response and accuracy [38].

The main aim of the current study is to evaluate and compare the significant driving behavior criteria between specified driving cultures (countries) by utilizing the FAHP framework. The Driver Behavior Questionnaire (DBQ) survey designed on the fuzzy scale is used to assess the responses of evaluators on perceived road issues. To better estimate the significance of the driver behavior criteria for road safety, the study analyzes twenty hypothetical driver behavior factors by pairwise comparison. A comprehensive FAHP approach is further used to assign weights to each examined factor and quantify the relative importance of each factor. Finally, the study highlights the most fundamental driver behavior factors solely related to road safety for each region. Meanwhile, Kendall’s coefficient of concordance was measured to show the degree of agreement between evaluator groups for each level.

## 2. Materials and Methods

### 2.1. Overall Workflow

To investigate the significant driver behavior criteria affecting traffic safety in this study, we use the following:

- finding the related criteria of DBQ and using in the questionnaires;
- designing required different levels of decision-making;
- applying the FAHP method for evaluating the criteria;
- evaluating the resulting weights by using Kendall’s agreement test.

The experimental outputs of the applied methods and their descriptions are organized in the following sections. Supplementary explanations and discussions about the significance of using the methods and criteria are represented in the discussion and conclusion sections.

### 2.2. Driver Behavior Questionnaire (DBQ) Characteristics

The Driver Behavior Questionnaire (DBQ) was the first developed approach to assess problematic driving behavior in the 1990s [39,40]. There has been a substantial effort made to detect and remediate behaviors that decrease driving safety. The Driver Behavior Questionnaire (DBQ) stands out for its longevity and dominant use among the many tools [41,42]. The previous study [43] identified three driving behavior types that included violations, lapses and errors, and investigated driving behavior and accident involvement relationship. The aberrant driver behaviors were studied using extended DBQ techniques which included ordinary and aggressive traffic violations, lapses and errors. Human error is defined as an unintentional decision or action, whereas violations are referred to as conscious decisions leading to failure. Slips and lapses occur without much conscious attention in very conversant tasks [44,45,46]. 

The study utilized the DBQ designed on a fuzzy scale to prioritize the significant driver behavior criteria for different traffic cultures. Car drivers having at least five-year driving experience were asked to fill the DBQ from designated countries such as Hungary, Turkey, Pakistan and China. DBQ survey data was collected by face to face method which enhanced its reliability. Furthermore, DBQ questionnaire data were collected from Turkey, Pakistan and China with the help of research assistants. While in Hungary, to collect DBQ data, the individuals were approached and asked to fill the DBQ survey. The questionnaire-based survey was divided into two portions: Demographic data were collected and their results (mean and standard deviation value) based on the driver’s response are shown in Table 1. We used digits (1, 0) for evaluation purposes to characterize driver occupation and gender. The second part of DBQ aimed to analyze the significant driver behavior criteria affecting road safety for different traffic cultures.

### 2.3. Significant Driver Behavior Criteria

**Figure 1:**
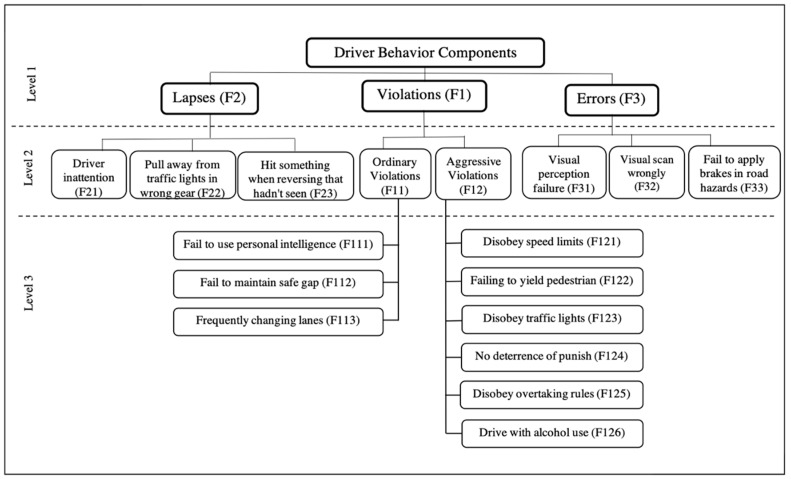
The hierarchical structure of the driver behavior criteria [47].

The study considered the well-acknowledged significant driver behavior criteria designed on the AHP framework [47] to compare and analyze the DBQ for different traffic cultures using FAHP. These driver behavior criteria influence road safety drastically and are also considered important for safe movements of other road users. Evans [48] claimed that how drivers behave is overwhelmingly the most critical factor determining overall traffic safety. For study purpose, the driver behavior criteria were designed in a three-level hierarchical structure and abbreviated alphabetically to evaluate each criterion comprehensively. The first level consists of main driver behavior criteria such as violations, lapses and errors. These main driver behavior criteria are divided into sub-criteria for level 2 and level 3 as shown in Figure 1.

### 2.4. Fuzzy Analytic Hierarchy Process (FAHP)

Various FAHP approaches and applications were used by different researchers. The first study in FAHP utilized triangular functions [49]. Cheng [50] introduced a new level analysis approach for the synthetic extent standards of the pairwise comparison for handling fuzzy AHP. The pairwise comparisons can provide a comparison scale to estimate the priorities in the hierarchical structure. FAHP modeling is an effective tool for decision making [51,52,53,54,55,56].

The FAHP method was applied in the current study which presents the determination of weights of driver behavior criteria and quantitative analysis of significant driver behavior criteria for different traffic cultures (China, Hungary, Pakistan and Turkey). The participants were asked to fill the driver behavior questionnaire designed on a fuzzy scale to better evaluate the driver behavior criteria affecting road safety. The designed hierarchical model was utilized for examining driver behavior and sub-criteria categorically by using fuzzy numbers based on pairwise comparisons (PCs). After applying the pairwise comparison on questionnaire survey data collected from evaluators of specified traffic cultures, the global scores were computed. In order to ensure the reliability of driver behavior data, the consistency check was performed. The authors briefly reviewed concepts for fuzzy hierarchical evaluation in this section.

The authors employed fuzzy logic by designing a questionnaire survey with a triangular fuzzy number as a pairwise comparison scale. The basic mathematics was used such as Refs. [57,58].

A fuzzy number $\tilde{T}$ on R to be a triangular fuzzy number if its membership function $\mu \tilde{T}\left(y\right):\;R\to \left[0,\;1\right]\;$ is equal to the following formula (1):

(1) $$\tilde{T}\left(y\right)=\{\begin{array}{c} \frac{y-1}{m-1},\;\;\;\;\;\;\;\;k\leq y\leq m\\ \frac{u-y}{u-m},\;\;\;\;\;\;\;\;m\leq y\leq u\\ 0,\;\;\;\;\;\;\;\;\;\;\;\;\;\;\;\mathrm{otherwise} \end{array}\;.$$

**Figure 2:**
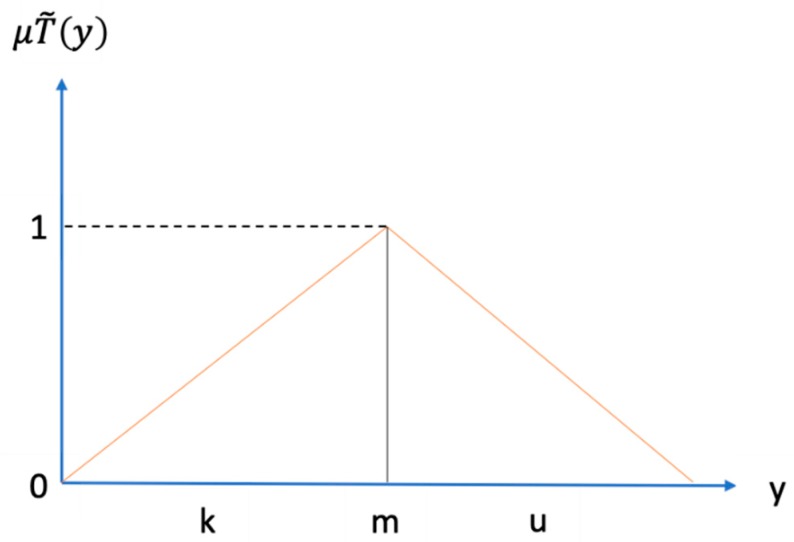
The triangular fuzzy set.

From formula (1), k and u mean the lower and upper bounds of the fuzzy number $\tilde{A}$, and m is the modal value for $\tilde{A}$ (like Figure 2). The triangular fuzzy number can be denoted by $\tilde{T}=\left(k,\;m,\;u\right)$. The operational laws of triangular fuzzy number $\tilde{T}{\;}_{1}=\left(k_{1},\;m_{1},\;u_{1}\right)$ and $\tilde{T}{\;}_{2}=\left(k_{2},\;m_{2},\;u_{2}\right)$ are displayed as the following Equations (2)–(6).

The addition of the fuzzy number ⨁

(2) $$\tilde{T}{\;}_{1}\;⨁\tilde{T}{\;}_{2}=\left(k_{1}+k_{2},m_{1}+m_{2},u_{1}+u_{2}\right),$$

Multiplication of the fuzzy number ⨂

(3) $$T{\;}_{1}\;⨂\tilde{T}{\;}_{2}=\left(k_{1}k_{2},\;m_{1}m_{2},\;u_{1}u_{2}\right)\;\mathrm{for}\;k_{1},\;k_{2}>0;m_{1},m_{2}>0;u_{1}u_{2}>,$$

Subtraction of the fuzzy number ⊝

(4) $$\tilde{T}{\;}_{1}\;⊝\tilde{T}{\;}_{2}=\left(k_{1}-u_{2},\;m_{1}-,m_{2},\;u_{1}-k_{2}\right),$$

Division of a fuzzy number ∅

(5) $$\tilde{T}{\;}_{1}\;\emptyset \tilde{T}{\;}_{2}=\left(\frac{k_{1}}{u_{2}},\frac{m_{1}}{m_{2}},\frac{u_{1}}{l_{2}}\right)\mathrm{for}\;k_{1},\;k_{2}>0;m_{1},m_{2}>0;u_{1}u_{2}>0,$$

Reciprocal of the fuzzy number

(6) $$\tilde{T}{\;}^{-1}=\left(\frac{1}{u_{1}},\frac{1}{m_{1}},\frac{1}{\;k_{1}}\right)\;\mathrm{for}\;\;\;k_{1},\;k_{2}>0;m_{1},m_{2}>0;u_{1}u_{2}>0.$$

In the current study, the computational technique is based on the triangular fuzzy numbers scale that was defined by [49] as shown in Table 2.

The employed pairwise comparison matrices are created based on the hierarchical structure (Figure 1). Pairwise comparisons are created by assigning linguistic terms to compare which criteria are the more significant than the other with respect to the main one, as T the bigger matrix (6×6) in the study as presented below:

(7) $$\tilde{T}=[\begin{array}{cccccc} 1 & {\tilde{a}}_{12} & {\tilde{a}}_{13} & {\tilde{a}}_{14} & {\tilde{a}}_{15} & {\tilde{a}}_{16}\\ {\tilde{a}}_{21} & 1 & {\tilde{a}}_{23} & {\tilde{a}}_{24} & {\tilde{a}}_{25} & {\tilde{a}}_{26}\\ {\tilde{a}}_{31} & {\tilde{a}}_{32} & 1 & {\tilde{a}}_{34} & {\tilde{a}}_{35} & {\tilde{a}}_{36}\\ {\tilde{a}}_{41} & {\tilde{a}}_{42} & {\tilde{a}}_{43} & 1 & {\tilde{a}}_{45} & {\tilde{a}}_{46}\\ {\tilde{a}}_{51} & {\tilde{a}}_{52} & {\tilde{a}}_{53} & {\tilde{a}}_{54} & 1 & {\tilde{a}}_{56}\\ {\tilde{a}}_{61} & {\tilde{a}}_{62} & {\tilde{a}}_{63} & {\tilde{a}}_{64} & {\tilde{a}}_{65} & 1 \end{array}]=[\begin{array}{cccccc} 1 & {\tilde{a}}_{12} & {\tilde{a}}_{13} & {\tilde{a}}_{14} & {\tilde{a}}_{15} & {\tilde{a}}_{16}\\ 1/{\tilde{a}}_{12} & 1 & {\tilde{a}}_{23} & {\tilde{a}}_{24} & {\tilde{a}}_{25} & {\tilde{a}}_{26}\\ 1/{\tilde{a}}_{13} & 1/{\tilde{a}}_{23} & 1 & {\tilde{a}}_{34} & {\tilde{a}}_{35} & {\tilde{a}}_{36}\\ 1/{\tilde{a}}_{14} & 1/{\tilde{a}}_{24} & 1/{\tilde{a}}_{34} & 1 & {\tilde{a}}_{45} & {\tilde{a}}_{46}\\ 1/{\tilde{a}}_{15} & 1/{\tilde{a}}_{25} & 1/{\tilde{a}}_{35} & 1/{\tilde{a}}_{45} & 1 & {\tilde{a}}_{56}\\ 1/{\tilde{a}}_{16} & 1/{\tilde{a}}_{26} & 1/{\tilde{a}}_{36} & 1/{\tilde{a}}_{46} & 1/{\tilde{a}}_{56} & 1 \end{array}],$$

where

$${\tilde{a}}_{ij}=\{\begin{array}{l} {\tilde{9}}^{-1},\;{\tilde{8}}^{-1},{\tilde{7}}^{-1},{\tilde{6}}^{-1},{\tilde{5}}^{-1},{\tilde{4}}^{-1},{\tilde{3}}^{-1},{\tilde{2}}^{-1},\tilde{1},\tilde{2},\tilde{3},\tilde{4},\tilde{5},\tilde{6},\tilde{7},\tilde{8},\tilde{9},\;\;\;1,i\neq j\\ 1,\;i=j \end{array},$$

where a ij is fuzzy comparison value of dimension i to criterion *j*.

For aggregating the fuzzy weights, the fuzzy geometric mean was used [55]:

(8) $${\tilde{r}}_{i}={\left({\tilde{a}}_{i1}\;⨂\;{\tilde{a}}_{i2}\;⨂{\tilde{\;a}}_{i3}⨂\;{\tilde{a}}_{i4}\;⨂\;{\tilde{a}}_{i5}\;⨂\;{\tilde{a}}_{i6}\right)}^{1/n},$$

(9) $${\tilde{r}}_{i}={\left({\tilde{a}}_{i1}\;⨂\;{\tilde{a}}_{i2}\;⨂{\tilde{\;a}}_{i3}⨂\;{\tilde{a}}_{i4}\;⨂\;{\tilde{a}}_{i5}\;⨂\;{\tilde{a}}_{i6}\right)}^{1/n},$$

$\mathrm{where}\;{\tilde{r}}_{i}$ is a geometric mean of fuzzy comparison value of criterion i to each criterion, ${\tilde{w}}_{i}$ is the fuzzy weight of the ith criterion which can be designated by a triangular fuzzy number, ${\tilde{w}}_{i}=\left(kw_{i},\;mw_{i},\;uw_{i}\right)$. The $kw_{i},\;mw_{i}\;\mathrm{and}\;\;uw_{i}$ stand for the lower, middle and upper values of the fuzzy weight of the ith dimension.

### 2.5. Kendall’s Agreement Test

The ranking of the factors is a very common need in engineering, management, education, finance, medicine and politics. Accordingly, the new positions, new products, new elections public or private services are ranked by the public, decision-makers and experts [47,59]. However, the basic question is how much the evaluated rankings are in concordance by different groups. To answer this question, the well-known method, Kendall’s coefficient of concordance (W), was introduced by Kendall and Smith in 1939 [60]. In addition, W is a normalization of the measurement of the Friedman test, which is studied as a non-parametric statistic method. Furthermore, it can be utilized for a set of criteria to measure the agreement level among different raters [61]. For the current study, the authors applied Kendall’s W procedure to estimate the agreement degree (the concordant degree) between different specified drivers’ groups for each level in the hierarchal structure. Kendall’s concordance degree (W) ranges from 0 (no agreement) to 1 (complete agreement. However, the values between 0 and 1 are interpreted in Table 3.

The calculation procedure starts by aggregating the ranking of factor i through the following equation:

(10) $$R_{i}=\overset{n}{\underset{j=1}{\sum }}r_{ij}\;,$$

where $R_{i}$ is the aggregated ranking of factor *i*, $r_{ij}$ is the rank given to factor *i* by the evaluator group *j*, *n* is the number of rater groups rating *m* factors. 

Then, calculating R, which is the mean of the $R_{i}$ values:

(11) $$R=\frac{n\left(m+1\right)}{2},$$

(12) $$K=\overset{n}{\underset{i=1}{\sum }}{\left(R_{i}-R\right)}^{2},$$

where *K* is a sum-of-squares statistic deviation over the row sums of ranking $R_{i}$. 

Following that, Kendall’s “W” statistic is between (0 and 1) and it can be measured from the following equation:

(13) $$W=\frac{12\;K}{n^{2}\;\left(\;m^{3}-m\right)}.$$

After implementing the equation, the outcome will give the concordance degree among the different observed groups.

## 3. Results

### 3.1. FAHP Ranking Results

**Figure 3:**
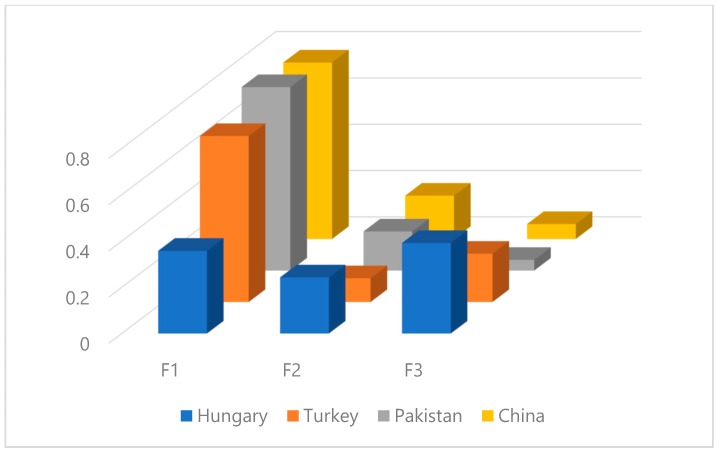
The global scores for evaluators from different regions in level 1.

The fuzzy analytic hierarchy process (FAHP) results are utilized to compare and prioritize the significant driver behavior criteria in a three-level hierarchical structure for different traffic cultures. For level 1, the study results found the same ranking of observed driver behavior criteria for three regions such as Turkey, Pakistan and China. Accordingly, violations (F1) is observed as the first rank criteria followed by lapses (F2) and errors (F3). The previous study also observed that road traffic violations (RTVs) are the most critical that cause certain risks to other road users [62]. However, Hungary drivers evaluated the errors (F3) as first rank criteria followed by violations (F1) and lapses (F2) as shown in Figure 3.

**Figure 4:**
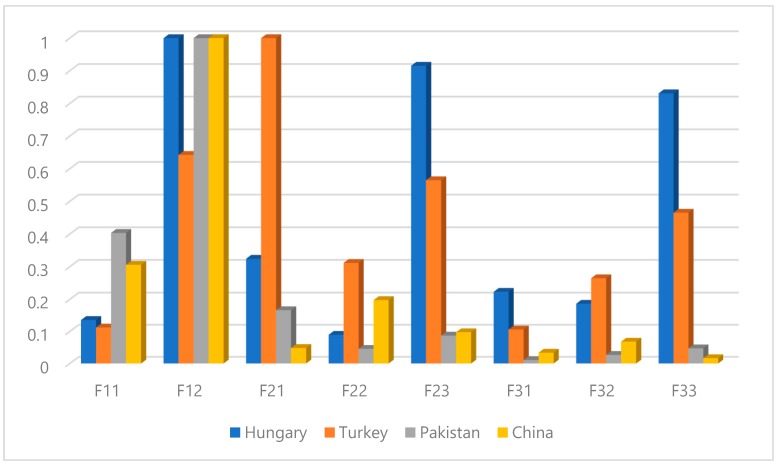
The global normalized scores for evaluators in level 2.

For level 2, the FAHP results enumerated the ranking of driver behavior sub-criteria for each region. The study results observed the aggressive violations (F12) as first rank criteria based on drivers’ responses from three regions such as Hungary, Pakistan and China. The previous study also found a significant relationship between aggressive violations and the number of accidents for Finland and Iran [7]. Meanwhile, Turkey drivers evaluated driver inattention (F21) as first rank criteria for level 2. Previous study results also evaluated driver inattention as the most frequent risky driver behavior [63]. Furthermore, ordinary violations (F11) is observed as second rank criteria from Pakistan and China drivers. However, Hungary and Turkey drivers enumerated the ordinary violations (F11) as the second least significant criteria for level 2. Moreover, visual perception failure (F31) is observed as the last rank criteria according to the point of view of Turkey and Pakistan drivers. Meanwhile, Hungary drivers evaluated the pull away from traffic lights in the wrong gear (F22) as last rank criteria and China drivers evaluated fail to apply brakes in road hazard (F33) as the last rank criteria. Similarly, different ranks are observed for other sub-criteria based on responses of evaluators from different traffic cultures as shown in Figure 4.

**Figure 5:**
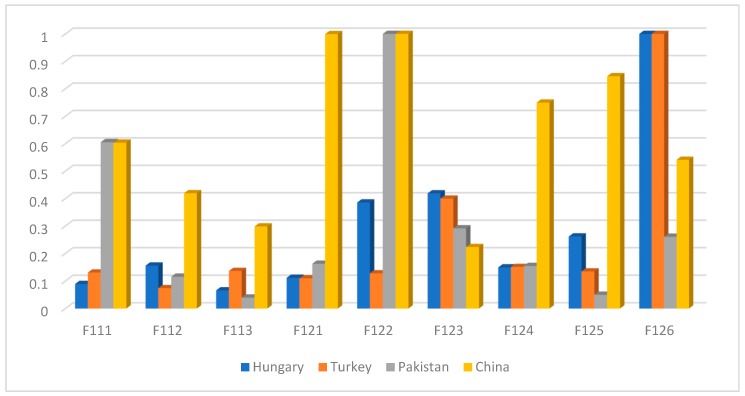
The global normalized scores for evaluators in level 3.

Finally, the study enumerated the ranking of sub-criteria for level 3 as shown in Figure 5. Results found that Pakistan and China drivers evaluated the fail to yield pedestrians (F122) as first rank criteria. The previous study detected in terms of contributing factors that 14.2% of fatalities were attributed to failure to yield right of way at the crossing [3]. The study noticed specifically the same ranking of all sub-criteria in this level for Hungary and Turkey drivers. Accordingly, the first rank criteria observed from Hungary and Turkey drivers is the drive with alcohol use (F126). According to Hungarian driving laws, there is a zero-tolerance policy towards drinking and driving [64]. Even with a small amount of alcohol use, drivers are twice expected to be involved in traffic accidents than moderate drivers [65]. Furthermore, Hungary and Turkey drivers enumerated the failure to maintain a safe gap (F112) as the last rank criteria. Meanwhile, Pakistan drivers evaluated the frequently changing lanes (F122) as the last rank criteria and Chinese drivers evaluated the no deterrence of punishing (F124) as the last rank criteria. The real traffic violation data in China showed that there are several repeat offenders among the multiple violation vehicles despite the application of a penalty point system [66].

### 3.2. Kendall’s Agreement Test Results

Kendall’s agreement test was performed to measure the concordance coefficient (W) between specified driver groups for each level of hierarchical structure. For level 1, Kendall’s coefficient results showed Kendall’s coefficient of concordance (W = 0.4375) between observed groups which represents a medium agreement but not a perfect agreement as shown in Table 4.

For level 2, Kendall’s agreement test was performed to measure Kendall’s coefficient of concordance between observed groups. The concordance value (W = 0.5446) showed medium agreement between specified groups as shown in Table 5.

For level 3, Kendall’s agreement test was also performed to measure Kendall’s coefficient of concordance between observed groups. The concordance value (W = 0.2354) showed a low agreement between specified groups as shown in Table 6.

## 4. Discussion

This study is investigative in providing a comprehensive analysis of the important driver behavior factors related to road safety for different driving cultures. The study results evaluated that each country has its own traffic safety issues related to driver behavior. The findings revealed “violations” as the most significant driver behavior criteria for level 1 from all designated regions except Hungary. While for Hungary drivers, “errors” is observed as the most significant criterion followed by “violations”. Furthermore, the FAHP results found “aggressive violations” as first rank criteria for level 2 from evaluators of Hungary, Pakistan and China. Meanwhile, Turkish drivers evaluated “driver inattention” as first rank criteria for level 2. A recent comparative study found that Turkey drivers with specific norms showed more errors and fewer positive behaviors [67]. However, the last rank criteria observed in level 2 for different countries are “ordinary violation” (Hungary and Turkey), “visual scan wrongly” (Pakistan) and “visual perception failure” (China). Furthermore, the study results found “fail to yield pedestrian” as the most significant driver behavior criteria for level 3 from Pakistan and China drivers. The previous study also noticed that the Chinese drivers tend not to decelerate to a full stop when passing an unsignalized intersection [68]. Meanwhile, Turkey and Hungary drivers evaluated the “drive with alcohol use” as the most significant driver behavior criteria for level 3. The last rank observed driver behavior criteria in level 3 for different regions are “fail to maintain safe gap (Hungary and Turkey), “frequently changing lanes (Pakistan) and “no deterrence of punishing” (China). Finally, Kendall’s agreement test was performed to measure the concordance coefficient (W) between specified driver groups for each level of hierarchical structure. The Kendall’s agreement test results showed medium agreement between observed drivers’ groups for level 1 and level 2. Meanwhile, for level 3, Kendall’s agreement test results showed weak agreement between observed groups. Finally, the study recommends that high-rank driver behavior factors should be focused on planning the road safety campaigns to improve the risk perception of road safety. In addition, the study recommends that high-rank risky driver behavior factors should be analyzed for different maneuver transition probabilities as utilized in Ref. [69] using driving simulators. Finally, for quality control of driving data, the Geographic Information Systems (GIS) could be useful for extracting driving patterns, and for detecting events, among others [70,71].

## 5. Conclusions

Human behavior is considered as complex and often uncertain in assigning the causes of road accidents by using conventional AHP. However, FAHP can overcome this problem by capturing the ambiguity of the human thinking style. Therefore, in the current study, a well proved multi-criteria decision-making method, the fuzzy AHP is used for comparison and prioritization of significant driver behavior criteria and sub-criteria among the different traffic cultures (Hungary, Turkey, Pakistan and China). We utilized the DBQ designed on the fuzzy scale for evaluation purposes based on PCs. The FAHP method measured the weights of driver behavior criteria and sub-criteria which enables us to rank these criteria in a three-level hierarchical structure for all specified regions. Meanwhile, Kendall’s agreement test was used to check the agreement degree between observed groups for each level of hierarchical structure. 

The FAHP study results highlighted the significant driver behavior criteria affecting road safety for different traffic cultures. All this important information could be useful to make the drivers aware of its own traffic risks for each country. Linkage of the observed data with traffic authorities may help to adopt effective local road safety strategies. FAHP method should be used in future research to analyze other significant road traffic elements and their sub-factors related to road safety such as road infrastructure, vehicles, traffic operators and environment.

## Figures and Tables

**Table 1:** Sample characteristics of participants.

Variables	Hungary	Turkey	Pakistan	China
*N*	70	70	70	70
**Age**				
Mean	25.61	26.87	29.31	27.41
SD	2.71	3.77	4.03	3.29
**Gender** (1 = male,0 = female)				
Mean	0.77	0.89	0.84	0.71
SD	0.41	0.48	0.52	0.31
**Driving Experience**				
Mean	5.29	7.07	8.73	6.57
SD	2.11	3.77	4.67	2.89
**Driver Occupation** (1 = job,0 = student)				
Mean	0.63	0.69	0.74	0.49
SD	0.37	0.41	0.46	0.23

**Table 2:** Triangular fuzzy numbers scale [58].

Fuzzy Number	Linguistic Variables	Triangular Fuzzy Numbers
9	Perfect	(8, 9, 10)
8	Absolute	(7, 8, 9)
7	Very good	(6, 7, 8)
6	Fairly good	(5, 6, 7)
5	Good	(4, 5, 6)
4	Preferable	(3, 4, 5)
3	Not bad	(2, 3, 4)
2	Weak advantage	(1, 2, 3)
1	Equal	(1, 1, 1)

**Table 3:** Kendall’s W agreement degree scale [61].

Correlation Coefficient	Interpretation
1	Perfect agreement
0.9–1	very high agreement
0.7–0.9	High agreement
0.4–0.7	Medium agreement
0.2–0.4	Low agreement
0–0.2	very low agreement
0	No agreement

**Table 4:** Kendall’s coefficient of concordance (W) for level 1.

Criteria	Hungary	Turkey	Pakistan	China	Ri	${\left(R_{i}-R\right)}^{2}$
F1	2	1	1	1	5	9
F2	3	3	2	2	10	4
F3	1	2	3	3	9	1
n = 4	m = 4	K = 14	R = 8	W = 0.4375		

**Table 5:** Kendall’s coefficient of concordance (W) for level 2.

Criteria	Hungary	Turkey	Pakistan	China	Ri	${\left(R_{i}-R\right)}^{2}$
F11	7	7	2	2	18	0
F12	1	2	1	1	5	169
F21	4	1	3	6	14	16
F22	8	5	6	3	22	16
F23	2	3	4	4	13	25
F31	5	8	8	7	28	100
F32	6	6	7	5	24	36
F33	3	4	5	8	20	4
n = 4	m = 8	K = 366	R = 18	W = 0.5446		

**Table 6:** Kendall’s coefficient of concordance (W) for level 3.

Criteria	Hungary	Turkey	Pakistan	China	Ri	${\left(R_{i}-R\right)}^{2}$
F111	6	6	2	6	20	0
F112	9	9	7	4	29	81
F113	4	4	9	6	23	9
F121	8	8	5	2	23	9
F122	7	7	1	1	16	16
F123	2	2	3	8	15	25
F124	3	3	6	9	21	1
F125	5	5	8	4	22	4
F126	1	1	4	5	11	81
n = 4	m = 9	K = 226	R = 20	W = 0.2354		
